# Knowledge and attitude towards child marriage practice among women married as children-a qualitative study in urban slums of Lahore, Pakistan

**DOI:** 10.1186/1471-2458-14-1148

**Published:** 2014-11-06

**Authors:** Muazzam Nasrullah, Rubeena Zakar, Muhammad Zakria Zakar, Safdar Abbas, Rabia Safdar, Mahwish Shaukat, Alexander Krämer

**Affiliations:** Department of Public Health Medicine, School of Public Health, Bielefeld University, Bielefeld, Germany; Institute of Social and Cultural Studies, University of the Punjab, Lahore, Pakistan; 2924 Clairmont Road, 30329 Atlanta, GA USA

**Keywords:** Child marriage, Women, Honor, Culture, Knowledge, Attitude, Pakistan

## Abstract

**Background:**

Child marriage (<18 years) is prevalent in Pakistan which is associated with negative health outcomes. Our aim is to describe women’s knowledge and attitude towards child marriage practice who themselves were married as children.

**Methods:**

Women of reproductive age (15–49 years) who were married prior to 18 years, for at least 5 years and had at least one child birth were recruited from most populous slum areas of Lahore, Pakistan. Themes for the interview were developed using published literature and everyday observations of the researchers. Interviews were conducted by trained interviewers in Urdu language and were translated into English. The interviews were tape-recorded, transcribed, analyzed and categorized into themes.

**Results:**

Nineteen of 20 participants who agreed to participate were married between 11–17 years. Most respondents were uneducated, poor and were working as housemaids. The majority participants were unaware of the negative health outcomes of child marriages. They appeared satisfied by the decision of their parents of marrying them before 18 years, and even condemned banning child marriages in Pakistan. Strong influence of culture and community perceptions, varying interpretation of religion, and protecting family honor are some of the reasons that were narrated by the participants, which seems playing a role in continuation of child marriage practice in Pakistan.

**Conclusion:**

Raising awareness of the negative health outcomes of child marriage, implementing and enforcing strict laws against child marriage practice, promoting civil, sexual and reproductive health rights for women, can help eliminate child marriages in Pakistan.

## Background

Around 100 million adolescent girls are expected to be married before the age of 18 years, also referred to as child marriage [1, 2] in the developing world during the next 10 years [3]. South Asia region has one of the highest rates of child marriage in the world [1], which is a clear violation of the Universal Declaration of Human Rights 1948 and the International Covenant on Civil and Political Rights 1966 [4]. Regardless of high maternal and child mortality and morbidity because of child marriage [5–9], the practice is highly prevalent in the region. Therefore, the United Nations highlight the need of reducing child marriage and its effect on maternal and child health through the Millennium Development Goals for improving maternal health, reducing child mortality, and promoting gender equality and women empowerment [10].

In Pakistan, where gender inequality is one of the highest in the world [11, 12], substantial number of girls are victims of child marriage [13]. Child marriage disproportionately affects females of poor, low-educated families residing in rural areas, and is associated with rapid repeat childbirth (<24 months apart), unwanted pregnancy, and pregnancy termination that predisposes young girls to maternal morbidity and mortality in Pakistan [13]. Child marriage was also found to be associated with increased likelihood of diarrhea, and under 5 mortality and infant mortality in Pakistan [14]. In particular, women married as children were found significantly associated with decreased likelihood of prenatal care and prenatal care by skilled medical care providers, and increased likelihood of delivery assistance by unskilled medical providers and delivery at home [15] that many a times resulted in medical complications such as postpartum hemorrhage [16].

Some of the known reasons for high prevalence of child marriages in Pakistan are poverty, traditional practices such as *WattaSatta* (bartering bride for bride), *PaitLikkhi* (marrying children before they are born or are still very young), *AddoBaddo* (marriage among tribes), and *Swara/Khoon-Baha/Vani/Sakh* (girls given in marriage as a form of dispute resolution), protecting the honor of child and family, and lack of implementation of legislation in Pakistan [4, 13, 15]. Factors such as income-earning activities and higher education are found to be protective factors of child marriage, however rigorous evaluations are lacking to determine whether these factors really work to prevent child marriage practice [1, 2, 17, 18]. Interventions become even harder to design when cultural practices are interwoven in societal norms, and without having insight about the knowledge and attitudes of girls and women about child marriage practice. To date there is less information available regarding what women think about child marriage practice who themselves were married as children, and what measures are needed to reduce this practice in the country. The aim of this study is therefore, to describe women’s knowledge and attitude towards child marriage practice who themselves were married as children in urban slums of Lahore, Pakistan.

## Methods

### Selection of participants

Data for this paper was drawn from in-depth interviews conducted with 19 pre-identified married women of reproductive age (15–49 years). Participants were selected for interview if the woman 1) was married before the age of 18 years (child marriage) 2) was married for at least 5 years, and 3) had at least one child birth. Reasons for setting the said selection criteria were to make sure that the interviewee had a prenatal and postnatal experience and that they have spent a considerable time in marital union. Participants were selected from urban slums of Lahore city, Pakistan. Lahore is the second most populous city in Pakistan with a population of over seven million [19]. According to the Population Association of Pakistan, 23.5% of the population of Lahore city is comprised of females of reproductive age, and 61% of women aged ≥15 years are married in the city [20]. We randomly selected six localities from two administrative towns with the most populous slum areas. Participants fulfilling the above-mentioned inclusion criteria were recruited from these localities by identification from a gatekeeper. Two gatekeepers who were employees of a non-governmental organization (NGO) in each of the selected towns helped the researchers in identification of the participants because of their efficient networking within the community. The NGO workers arranged the interview time and place as per convenience of the participants.

A total of 20 participants were approached by the researchers, of which 19 agreed to participate. The interviews were conducted during a period of 16 weeks (June to September 2013). The majority of interviews (52.6%; 10 of 19) were conducted at the participants’ homes when they were alone during the daytime. The remaining 9 interviews (47.4%) were conducted at the NGO workers’ office because of privacy and convenience of the participants.

### Data collection

Themes for the interview were developed using published scientific literature and everyday observations of the researchers. The in-depth interview guide was drafted and revised a few times by the researchers, and later field tested on two participants. The guide was further revised based on the feedback received from these two participants. Data collection was conducted by two well-trained and experienced interviewers (RS, MS). The interview time varied in length from one to one and a half hour.

The interviews were open-ended and carried out in a conversational style. In-depth semi-structured face-to-face individual interviews were conducted and transcribed in a separate room to maintain the privacy of the participants. The researchers established a rapport with participants for 10–15 minutes before the start of each session by sharing their respective experiences of married life. Each interview was followed by a 10 minutes closing casual conversation. Field notes and casual encounters with participants were also noted by the interviewers during the interview.

### Ethical considerations

Respondent’s safety, privacy and anonymity was maintained during the recruitment and interviews of the participants as described in the World Health Organization guidelines [21]. The procedure of consent was also followed as described in the guidelines. Written informed consent was obtained after the interviewer explained the participants, the aims, objectives, themes of discussion, researcher’s background, and details of the project. The interviews with the participants were voluntary and they were allowed to discontinue the interview at any time. During the interview, no personal information like addresses and phone numbers were collected. Names of the participants were converted into unique dummy names for the purpose of analysis. The study methodology was reviewed and approved by Institutional Review Board (IRB) of University of the Punjab, Lahore, Pakistan (reference number: D/688/FBSS).

### Data management and analysis

In-depth interviews were conducted in Urdu language, as most of the participants were illiterate and were not well-versed with English. The note-taking was also done in Urdu language. All of the interviews were tape-recorded with the permission of the participants. Urdu transcripts were then translated into English and then back translated for accuracy and quality of translation by one of the interviewer. All of the transcribed interviews/ discussions, memoranda, and field notes were entered into Microsoft Excel. A coding system was organized and themes were manually interpreted. A scheme of numbers and letters were used to designate major categories and subcategories. Hard copies of all computer files of data were also coded using colored pens to mark the margins with appropriate numbers and letters, whenever needed. Transcripts were then analyzed and categorized into themes.

## Results

### Participant characteristics

The age range of the participants was 21 to 34 years old. All of them were married between the ages 11 and 17 years. Almost all participants (n = 18; 94.7%) were married at the time of interview and were living with their husbands or in joint/extended families with their in-laws. Only five percent (n = 1) of participants were separated and living with their daughters at the time of interview. The majority of the respondents (n = 11; 57.9%) were migrants to the urban city from rural areas and the rest of eight (42.1%) respondents belonged to the urban setting. Based on the occupational categories of the respondents and their husbands, fifteen respondents (78.4%) belonged to a low socio-economic class. Four of the respondents belonged to middle socio-economic class and were either housewives or working as school teachers. The majority of the respondents (n = 13; 68%) were uneducated. About 15.8% (n = 3) respondents had up to primary education, and the remaining 15.8% (n = 3) had secondary education. More than half of the respondents (57.9%) were working as housemaids and the remaining (n = 8; 42.1%) were housewives.

### Perceptions about child marriage practice and awareness of its negative health outcomes

A majority of the participants (13 of 19) were not aware of the negative health outcomes of child marriages. However, over a quarter of participants (5 of 19) believed that negative outcomes of child marriages were not only confined to medical grounds but that they affected the social relations negatively. These participants (5 of 19) also reported a number of health problems, which included frequent pains, disturbed menstrual cycle, abortion, difficulty in child birth and physical weakness. The women narrated that they suffer from more health problems than those in their social circle who were married after the age of twenty. Despite these health problems, the participants were unaware of the negative health outcomes of child marriages and whether these health problems might be due to their early marriages. The participants felt that they were not mature enough to handle delicate matters of child health and child bearing. The women also felt guilty that they were not capable of handling the customary obligations of married life within families and faced social stigma.

Parents should not marry their daughters before the age of 20 because before 18, girls are not physically and mentally prepared for marriage. [Participant in early twenties, married before 18 years with secondary education]

However, the majority of women married as children (13 of 19) was satisfied and felt that their parents made the right decision of marrying them prior to 18 years of age.

I find myself very lucky because I got such a nice, sober and caring person. I believe that girls should be married at younger age provided that there is good proposal. [Participant in early thirties, married at the age of 12 years with five years of schooling]

In contrast, a sizeable number of women (6 of 19) strongly condemned child marriages. Participants mentioned that girls must be given an opportunity to complete their education before their marriage, “Educating a daughter means educating the whole family”. Few women (4 of 19) were of the view that if women are married before 18 years, they would not be able to complete their education. Furthermore, they would have less skills and competencies to care, educate and up-bring their children properly. Thus they would be unable to contribute positively to the family affairs.

I think that early marriages most often result in conflicts, divorce, ‘husband’ violence, and health issues for the females. Those girls who get married in later age are better ones…and I think girls should be allowed to complete their basic schooling first, then they should be married. [Participant in early twenties, uneducated, married at the age of 13 years]

### Attitude towards child marriage practice

#### Intention to marry participants’ daughters before 18 years

More than half of the participants (10 of 19) narrated that they would marry their daughters before the age of 18 years subject to the availability of a good marriage proposal. Moreover, participants provided religious justification in favor of their view-point. They believed that according to the teaching of Islam it was parents’ duty to marry their daughters as soon as they reach puberty. The participants mentioned a greater success and better adjustment with the extended family members if women are married before the age of 18 years.

As I said earlier, girls should be married early. It is easier for them to adjust in their ‘own’ (new) home at a younger age. Women of older age usually cannot adjust with in-laws [because of their maturity] and they suffer more. [Participant in early thirties, educated till 10th grade, and married at the age of 16 years]

Only four of 19 respondents were against the marriage of their daughters before the age of 18 years. These women wanted their daughters to get education and better social status in the society.

I have witnessed so many hardships and troubles just because of my child marriage. I will never let my daughters face those hardships and will not marry them in their childhood. Instead, I will educate them and try my utmost to give them better life and future. [Participant in late twenties, without any formal schooling, and married at the age of 12 years]

#### Opinion of participants whether child marriage should be continued in Pakistan

Almost half of the participants (10 of 19) were in favor of child marriage practice in Pakistan, and justified the practice by narrating reasons such as better adjustment of younger girls to in-laws, avoiding social evils, delinquency, and immorality [adultery]. According to the participants, not marrying the children before 18 years could result in situations which can cause social evils, sins, and social problems.

Access to mobile and TV cable has made the younger generation more vulnerable to many social evils. I have seen some cases where unmarried girls come to our clinic and ask for abortion of their illegal child. I am afraid of *moasharti bay rahrwi* [social evils] and I will marry my daughter [before 18] as soon as I find any suitable proposal for her. [Participant in mid-thirties, uneducated, and married at the age of 15 years]

Few participants (3 of 19) had the opinion that parents should evaluate the boy and his family before marrying their daughters, and at the availability of a good proposal, parents should marry their daughter before the age of 18 years.

It [child marriage] is a tradition and it should be continued. But, people are so strange that they marry their daughters without any investigation about a proposal. I think so it is the *Farz* [duty] of parents to check the boy’s character, his earning status and his family thoroughly before marrying their daughters. Otherwise, it is better that the daughter stays unmarried all her life rather than to be married to an irresponsible person. [Participant in early twenties, educated till 10th grade, and married at the age of 13 years]

In contrast, some participants (6 of 19) had the view that girls should not be married before 18 years. Necessary education and training is a pre-requisite to lead an independent life otherwise, they would face much difficulties and challenges in their married life.

“Girls should be allowed to express their choice of *Shareeq e hayat* [life partner] and if that proposal seems good, she should be married but preferably not before the age of 16. [Participant in early forties, uneducated, married at the age of 13 years]

Those women who favored the idea of child marriage condemned banning child marriages in the country. A vast majority of women viewed it purely a family matter in which the state should not interfere, whereas other believed that the state should make and implement strict laws to prohibit child marriages.

## Discussion

Over a quarter of participants in our study narrated that they suffered from several health problems such as frequent pains, disturbed menstrual cycle, abortion, difficulty in child birth and physical weakness, which were more than those in their counterparts who were married after the age of twenty. Although these health problems may be a direct consequence of low socio-economic status with lack of money to afford the expensive healthcare or to maintain a good diet, nonetheless it is evident from prior research within Pakistan and its neighboring countries, that child marriage was found to be associated with negative fertility-control outcomes [13, 22, 23], and child diarrhea and malnutrition [14, 24] even after controlling for social vulnerabilities such as women’s economic status, education, ethnicity, and place of residence. This makes us ponder whether some of the cultural factors and attitude towards child marriage practice, unlike social vulnerabilities, are playing a role in the continuation of child marriage practice in Pakistan. Controlling behavior of husbands and in-laws towards women married as children [1, 2, 9, 25], limited power of women for health-related decisions within the household, and lack of education and media exposure [26], especially in rural areas may explain why women in our study were not aware of negative health outcomes of child marriages. It was rather worrisome that the majority of women married as children in our study was satisfied and felt that their parents made the right decision of marrying them as children. It was even more disturbing that the majority of the women in our study condemned banning child marriages in the country, and viewed this purely a family matter. The more rigid sex-role stereotypes, and the patriarchal non-egalitarian expectations directed towards women may explain the reason why most women in our study felt satisfied with the decision of their parents. However, in-depth research is needed to understand the cultural- and behavioral-specific reasons for the continuation of child marriage practice and gender role in Pakistan.

The majority of the participants in our study narrated their willingness to marry their daughters before the age of 18 years subject to the availability of a good marriage proposal. The participants tried to justify their view-point with the connotation that the religion of Islam also persuades parents to marry their daughters as soon they attain puberty. The role of religion and religious leaders is profound in Pakistan with several religious leaders having strong hold in their communities [4]. This strong influence of religious leaders may affect the willingness of parents to marry their children at much younger age. Further, varying interpretation of religion may also play a role in the continuation of child marriage practice in the country. Shariah Law defines puberty/menstruation for girls and facial hair for boys to signify the time when they can get married. However, because of lack of awareness and limited knowledge, especially in the rural areas where most people are uneducated, the mandatory condition in Shariah of having mutual consent of both partners in marriage is often overlooked, which is the case most often in child marriages [27]. Secondly, some religious leaders have the opinion that both physical and psychological maturity is important before one can get married [4]. While most parents only know the aspect of physical maturity, psychological mental maturity that is required in Islam is usually ignored by parents while deciding about the marriage of their daughters [4]. Thirdly, historically transmitted powerful influence of patriarchal ideology continues to reinforce custom and traditions such as child marriages which put women in subservient position to men.

Most participants justified the practice of child marriage by giving reasons such as avoiding social evils, delinquency, and adultery. According to the participants, not marrying the children before the age of 18 years could result in situations which can cause social evils, sins, and social problems. Protecting the “family honor” is listed as one of the reasons for child marriages in earlier studies [4, 27]. The moment girls reach puberty, they are believed to be a source of attraction and lust for boys, parents thus, feel relaxed and free of burden of guarding their girls from unchastely by marrying them at an early age. By marrying girls before the age of 18 years, parents believe that this practice could protect their daughters from unwanted attention from men and likelihood of objectionable romantic relationship. Further, dropping out of girls from schools in the name of protecting family honor is a unfortunate consequence, because parents are often afraid of socially undesirable happenings at schools once their girls reach puberty [4]. According to an estimate in the year 2000, only 25% of women were able to complete their primary education as compared to 49% of men in Pakistan [12].

Pakistan faced several challenges in dealing with the issue of child marriage practice. The Child marriage Act Restraint 1929 prohibits the marriages of children below the age of 16 for girls and 18 for boys [28]. However, through the Child Marriages Restraint (Amendment) Bill 2009 and the Charter of Child Rights Bill 2009 efforts have been made in the country to increase the age of marriage to 18 years for girls. These efforts will help eliminate, at least on paper, the discriminatory provisions of age and aligning the legislation with the requirements of international laws against child marriages such as Convention on the Rights of the Child 1989 [4]. Further, primitive traditional practices such as *Watta Satta*, *Pait Likkhi*, *Addo Baddo*, and *Swara/Khoon-Baha/Vani/Sakh* are still prevalent in Pakistan [27], contrary to some of the country’s laws against them. These deep-rooted cultural practices needs significant efforts at local and governmental level to abolish them, which may help reduce child marriage practice in Pakistan.

There are several limitations of this study that should be seen in context of the overall paucity of information on child marriages in Pakistan. Findings presented in this paper are based only on interviews of 19 pre-identified married women of reproductive age who had been married before the age of 18 years, and are therefore not generalizable to the views of all women in Pakistan. Further, participants were recruited from most populous slum areas of Lahore, and may not be representative of other localities and cities of Pakistan. Findings can be subject to recall and social desirability biases even though we tried to reduce these biases by conducting interviews either at the participants’ homes when they were alone during the daytime or at the NGO workers’ office to give participants a more private opportunity to report the sensitive information. Most of the participants in our study were uneducated that may have introduced a bias in the study. The health problems reported by the participants may be a direct consequence of their low socio-economic status by lacking their ability to afford the expensive healthcare or to maintain a good diet, therefore the findings need to be interpreted with caution. However, previous studies have shown the negative health outcomes among those married as children even after controlling for social vulnerabilities such as women’s economic status, education, ethnicity, and place of residence [13, 14, 22–24].

## Conclusion

A majority of the participants were unaware of the negative health outcomes of child marriages. Most women favored child marriage practice and intended to marry their daughters before the age of 18 years. The participants were satisfied by their parent’s decision of marrying them before the age of 18 years, and condemned banning child marriages in the country. Strong influence of culture and community perceptions, varying interpretation of religion, and protecting family honor are some of the factors that may play role in the continuation of child marriage practice in Pakistan. Raising awareness of the negative health outcomes of child marriage [13–15] by the government, local and international NGOs, implementing and enforcing strict laws against child marriage practice, promoting civil, sexual and reproductive health rights for women, and provision of economic opportunities for girls and their families such as microfinance schemes can help eliminate child marriage practice in Pakistan.
